# Imagination

**Published:** 1890-04

**Authors:** J. R Callahan

**Affiliations:** Cincinnati, O.


### THE DENTAL REGISTER.

VOL. XLIV.] APRIL, 1890. [No. 4.

### COMMUNICATIONS.

### Imagination.

BY J. R CALLAHAN, D.D.S., CINCINNATI, O.

Read before the Odontological Society of Cincinnati.

Since I promised our worthy President to write a paper on this subject I have regretted my rash promise more than once. First, the title is misleading. It should be, "Influence of the Mind upon the Body." Second, the subject is beyond my calibre. To write upon this subject, one should be prepared by years of special study, and then it would require a volume to express a well developed idea, instead of a short paper. But, if I can simply direct your attention to this somewhat neglected field I will have performed a good work. Most of the ideas and illustrations I use here I have gathered from numerous books I have read on the subject and from lectures I have attended this winter.

For our present purposes the term "Imagination" signifies, in popular and medical language, that a man imagines certain bodily phenomena to have occurred which have not; or, it is meant that certain bodily phenomena which really have occurred, owe their existence solely to the influence of his imagination or image-making power. This short definition of the term would be very unsatisfactory to a person who has any desire to give the subject an exhaustive study. There are many long drawn out, and tedious efforts to get at or expound and separate the emotional and the ideational states of the mind, and to show the close relation and influence of expectation, belief, faith, imitation, sympathy, hope, etc. These definitions are beautifully expressed by such men as Herbert Spencer, Mr. Ruskin and others. Webster uses the following lines from Shakespeare:

The lunatic, the lover and the poet
Are of imagination all compact.
The poet's eye, in a fine frenzy rolling,
Doth glance from heaven to earth, from earth to heaven.
And as imagination bodies forth
The form of things unknown, the poet's pen
Turns them to shapes, and gives to airy
Nothing a local habition and a name."

As I have said before, our short definition will answer our purpose to-night, viz.: That certain bodily phenomena which really have occurred owe their existence solely to the influence of the imagination or image-making power. When a person on swalowing a bread pill in belief that it possesses apparient properties is purged, it is said to be through his imagination. The mental condition present, yielding on analysis a definite direction of thoughts to the intestinal canal, such leading idea exciting the same peristallic action as would have been induced by castor oil. The force of this current of thought is augumented by expectations, in such cases. The fixed idea is, that certain phenomena will occur in such cases; we say, it is due to imagination.

That the imagination plays an important part in every day life I think no one will question; that it plays an important role in our every day practice I firmly believe. Certain conditions may be brought on by imagination, certain pathologic conditions may also be corrected by the imagination.

The intellect may excite ordinary sensation (æsthesia), which in addition may be either excessive (hyperæsthesia) or diminished (anæsthesia), etc. The comprehensive expression of John Hunter contains in a nut-shell the principal which underlies the greater part of the phenomena referred to. He says, I am confident that I can fix my attention to any part until I have a sensation in that part. It is a very common experiment to have persons to make pressure on the knee, or any other convenient part of the body, with the index finger or thumb, at the same time direct the whole mind or attention on that point for a few minutes, when a pain or sensation will appear at that point.

When a person has that control of the mind, and can control it to that degree, he is said to be under control, and any person who is under control can hypnotize or be hypnotized.

The imagination then has great influence on sensation. You have often known of a person's teeth being set on edge by witnessing another about to pass a sharp instrument over glass or porcelain; also the production of shuddering by the mentioning of objects which, if present, would excite that sensation. That is by recollective imagination. I can not think of seeing a slate rubbed with a dry sponge, says Herbert Spencer, without there running through me the same thrill that actually seeing it produces. As to hyperæsthesia, it is unnecessary in an assembly of dentists to do more than to refer in illustration to the fact that the expectation of pain greatly intensifies it; yet is in short, the converse of the other truth, that pain is not felt, or only slightly felt, when the attention is directed in a different direction. Anæsthesia, insensibility to bodily pain artificially induced without drugs and solely by physical means, is a most interesting and important fact, and worthy of our most profound study. We will leave hypnotism to a more convenient season.

Mr. Woodhouse Braine, of London Cross, gives a striking case of imaginary anæsthesia: He says, on June 26, 1872, Kate Levy, aged 20, came to the Dental Hospital of London, to have some carious teeth extracted. As more than one sitting was deemed necessary, it was proposed to remove the two most painful and difficult teeth, and then order her to come again. The patient was of a very hysterical temperament, but had nitrous oxide administered without any difficulty, and the extractions were performed. When she came to herself she refused to sit up in the chair, or to push the piece of wood from between her teeth. She remained motionless, not taking any notice of surrounding objects, and not doing anything she was told to do. That she was conscious I knew by the expression of her face, by the quivering of both eyelids and by the reflex action which immediately ensued on touching the conjunctiva. I therefore said to the operator: if she is still unconscious there will be time to remove another tooth (for eleven teeth and roots had to be extracted), and to my surprise this was done without any apparent suffering. I then remembered previous cases of this nature, and said to the operator: Now she is coming round. She thereupon opened her eyes, sat up and recovered in the usual way. She came again on June 21, and wishing to try the influence her imagination had on her sensibility, I determined to administer air only. She breathed this for a few seconds, and on my calling the pupils' attention to her quiet breathing, she began to inhale the air more deeply, and then on my giving her the cue, by saying to the pupils present: Now, you see when I lift up her hand and let it go it will fall heavily. It turned out as was predicted. Then saying: Now, you will find she will breathe rapidly and then cease to feel pain, although she will know something is being done. I removed the face-piece. The operator then commenced his extractions—removed four teeth. In the hospital note-book is written by the surgeon on duty that day. Note.—The patient breathed air only through the inhaler. One firm tooth, two firm stumps and a temporary tooth were extracted. Said she felt no pain, but felt the teeth coming out—a well marked case of hysteria. This patient came again, by order, on June 29, and on several other occasions, when air only was administered, and each time the same psychical anæsthesia was apparent.

In regard to the special senses the influence of the mind is notorious. The influence upon the olfactory sense is well exhibited in a case reported by Bennett, who says: A clergyman told me that some time ago suspicions were entertained in his parish of a woman who was supposed to have poisoned her newly born infant. The coffin was exhumed and the procurator fiscal, who attended with the medical men to examine the body, declared that he already perceived the odor of decomposition, which made him feel faint, and in consequence he withdrew. But on opening the coffin it was found to be empty, and it was afterward ascertained that no child had been born, and consequently no murder committed.

As regards the sense of hearing, it is very manifest that the thought uppermost in the mind makes a real sensation from without assuming a different character. If two children listening to a peal of bells, one is told they say long live the king, and the other never-forever, to each the chimes may sound as he expects to hear it.

So with sense of taste, with imaginative people. The food eaten or the fluid drunk assumes a very different taste according to the fancy. I once knew a gentleman, at a hotel in this city, to complain bitterly of the meat being tough and not fit to eat. The servant removed the meat and in a few minutes brought back the same piece of meat he had taken out, and my friend ate it with great relish, and gave the waiter a quarter for his kind attention. I learned from the steward that nothing had been done to the meat only keeping it warm, and furthermore that it is a very common practice, and is thought to be an easy way to satisfy constitutional kickers, as they are called by hotel people.

So without limit, almost, we might go on and give examples of the influence of the imagination upon the different senses; upon muscles, voluntary and involuntary; upon the heart, temperature, alimentary canal, in fact, any part or function of the body.

Out of what little I have said is there not food for reflection. I will stop here, just on the threshold of the subject, and leave you, as dentists, to make the practical applications of what little we know of imagination.

